# The fewer, the better fare: Can the loss of vegetation in the Cerrado drive the increase in dengue fever cases infection?

**DOI:** 10.1371/journal.pone.0262473

**Published:** 2022-01-13

**Authors:** Arlindo Ananias Pereira da Silva, Adriano Roberto Franquelino, Paulo Eduardo Teodoro, Rafael Montanari, Glaucia Amorim Faria, Cristóvão Henrique Ribeiro da Silva, Dayane Bortoloto da Silva, Walter Aparecido Ribeiro Júnior, Franciele Muchalak, Kassia Maria Cruz Souza, Marcos Henrique Prudencio da Silva, Larissa Pereira Ribeiro Teodoro

**Affiliations:** 1 Graduate Program in Agronomy–Cropping Systems, São Paulo State University (Unesp), Ilha Solteira, SP, Brazil; 2 Graduate Program in Geography, São Paulo State University (Unesp), School of Technology and Sciences, Presidente Prudente, SP, Brazil; 3 Federal University of Mato Grosso do Sul (UFMS), Chapadão do Sul, MS, Brazil; 4 Federal University of Acre (UFAC), Rio Branco, AC, Brazil; 5 Graduate Program in Agronomy, State University of Londrina (Uel), Londrina, PR, Brazil; 6 Graduate Program in Sciences—Nuclear Energy in Agriculture, Centro de Energia Nuclear na Agricultura, São Paulo University, Piracicaba, Brazil; 7 São Paulo State University (Unesp), Engineering School, Ilha Solteira, SP, Brazil; 8 Graduate Program in Geography, Federal University of Mato Grosso do Sul (UFMS), Três Lagoas, MS, Brazil; University of Brasilia, BRAZIL

## Abstract

Several studies have reported the relationship of deforestation with increased incidence of infectious diseases, mainly due to the deregulation caused in these environments. The purpose of this study was to answer the following questions: a) is increased loss of vegetation related to dengue cases in the Brazilian Cerrado? b) how do different regions of the tropical savanna biome present distinct patterns for total dengue cases and vegetation loss? c) what is the projection of a future scenario of deforestation and an increased number of dengue cases in 2030? Thus, this study aimed to assess the relationship between loss of native vegetation in the Cerrado and dengue infection. In this paper, we quantify the entire deforested area and dengue infection cases from 2001 to 2019. For data analyses, we used Poisson generalized linear model, descriptive statistics, cluster analysis, non-parametric statistics, and autoregressive integrated moving average (ARIMA) models to predict loss of vegetation and fever dengue cases for the next decade. Cluster analysis revealed the formation of four clusters among the states. Our results showed significant increases in loss of native vegetation in all states, with the exception of Piauí. As for dengue cases, there were increases in the states of Minas Gerais, São Paulo, and Mato Grosso. Based on projections for 2030, Minas Gerais will register about 4,000 dengue cases per 100,000 inhabitants, São Paulo 750 dengue cases per 100,000 inhabitants, and Mato Grosso 500 dengue cases per 100,000 inhabitants. To reduce these projections, Brazil will need to control deforestation and implement public health, environmental and social policies, requiring a joint effort from all spheres of society.

## Introduction

Infectious diseases in the world were overshadowed in 2020 by Coronavirus disease (COVID-19), decreed on March 11, 2020, by the World Health Organization—WHO [1] as a pandemic. Some diseases have been neglected worldwide by the media and governments, have high contagion rates and lethality, being a global problem for public health. The number of cases has increased in recent years. In 2019, for example, the WHO reported that 219 million people were affected by malaria and 10 million people were affected by tuberculosis there were around the world, [2] and, to a lesser extent, but just as worrying, the cases of dengue in 2020 reached approximately 2.7 million people. Of these, 36.5% were concentrated in Brazil, and more than half of these cases were recorded in the Cerrado biome [3].

The vector of dengue, the *Aedes aegypti* mosquito, originates from the African continent [4]. Its advance occurs in tropical areas due to the rapid increase in the urbanization process, especially in cities without basic sanitation infrastructure or in precarious conditions, thus increasing the occurrence of this arbovirosis. Dengue is a benign or severe acute febrile infectious pathology transmitted by the bite of infected mosquitoes (females), and is considered a major public health problem due to the increased number of cases and high lethality rate. From 2008 to 2019, dengue accounted for 6,429 deaths and currently presents a disease burden of 66,420 confirmed cases in the year 2021 in the Cerrado biome [3].

From the 1960s on, there has been a growing occupation of the Cerrado stimulated by agriculture [5], and as a consequence occurred a huge deforestation of this biome over the years. Since the beginning of its occupation, the Cerrado has accumulated a 31.20% loss of original native vegetation. Since 2005, there has been a reduction in deforestation, but in 2020 there was an increase of 13.21% compared to the previous year [6]. The Cerrado biome is increasingly threatened by the lack of government policies to combat deforestation [7]. The loss of vegetation in this region is related to human actions and occurs in greater proportions in areas where there is agricultural activity with more access to roads and urban areas. This is the result of land speculation for the production of commodities [8].

Even though Brazil has the greatest biodiversity in the world, it is one of the four tropical countries with the largest deforested areas [9, 10]. The current scenario is worrying, in which the loss of native vegetation causes several problems to the populations, which can mention the increase in the number of virus infections from vectors [11–13], since deforestation directly affects ecological relations in these environments [14, 15], making the number of mosquito predators to decrease due to lack of habitat and improved environmental conditions for vector development (e.g. increased temperature and food availability). And indirectly causes damage to public health [16, 17]. Several studies have pointed out a positive relationship between the increase in cases of human infection by viruses from vectors, whether vertebrates or not, in regions of tropical forests [13, 18–21]. However, few studies consider this topic in savanna regions. This study started from the hypothesis that there is a positive relationship between deforestation and the increase in dengue cases.

In this way, this study sought to answer the following questions: a) is the increase in vegetation loss related to dengue cases in the Brazilian Cerrado? b) how do different regions of the same tropical savanna biome show different patterns for the total cases of dengue and loss of vegetation? c) what is the forecast for a future deforestation scenario and the increase in the number of dengue cases in 2030?

## Methods

### Study design

The Cerrado biome has been indicated as one of the 25 global biodiversity hotspots for priority conservation [7]. Its flora, with more than 6,000 species of vascular plants [22], has a large number of endemic species [23]. It is characterized by presenting several phytophysiognomies, from forest to savanna and grasslands formations, the latter presenting more dynamic characteristics considering a short period [24].

**Fig 1:**
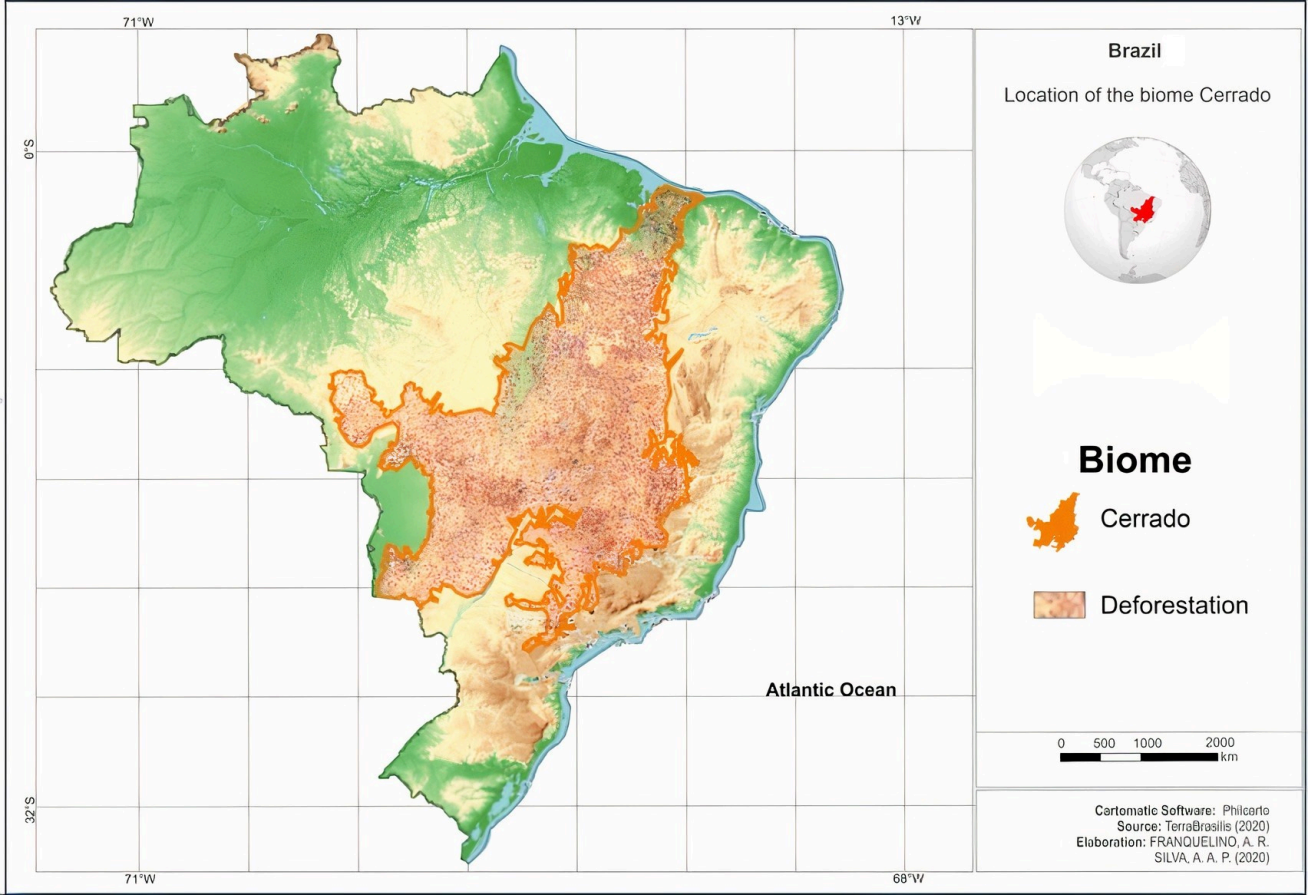
Delimitation of the Cerrado biome throughout the national territory and estimates of the loss of native vegetation (km²). Corel Draw was used in the image preparation with km² of loss of native vegetation data available through the TerraBrasilis platform (http://terrabrasilis.dpi.inpe.br/app/map/deforestation). Deforestation indicates the area deforested in the Cerrado Biome between the years 2001 and 2019.

As it occupies the central portion of Brazil, the Cerrado biome borders on all the other biomes, except the Pampa. It is present in the states of Goiás, Tocantins, Bahia, Maranhão, Mato Grosso, Mato Grosso do Sul, Minas Gerais, Piauí, and São Paulo, including the Federal District. It presents forest and grasslands formations, and in these, the Savanna is the most expressive, having the most common physiognomy, the grasslands with sparse trees and shrubs, on grass carpets [25]. Fig 1 shows the delimitation of the Cerrado throughout Brazilian territory and the estimates of the loss of native vegetation (km²).

### Ecological study and data

Our study area is the Cerrado biome and the states that comprise it: Goiás, Tocantins, Distrito Federal, Bahia, Maranhão, Mato Grosso, Mato Grosso do Sul, Minas Gerais, Piauí, and São Paulo, and the years from 2001 to 2019. The probable cases of incidence of dengue fever cases per 100,000 inhabitants was obtained from the Ministry of Health website (https://www.gov.br/saude/pt-br/assuntos/boletins-epidemiologicos-1/) by epidemiological reports made available by the state health secretariats and compiled by the Brazilian Ministry of Health. As the study states have more than one biome, except GO and DF, municipalities that contain the Cerrado biome along its boundary were filtered and considered in analysis. It is worth noting that the data on dengue infection comes from the notifications made by the state health departments to the federal agencies. Thus, underreporting can occur, since the population often does not seek medical care, in addition to the occurrence of asymptomatic cases.

Annual data on loss of native vegetation and cases of dengue fever were acquired between 2001 and 2019. Data on the loss of native vegetation in the Cerrado were downloaded directly from the government website TerraBrasilis/INPE (http://terrabrasilis.dpi.inpe.br/app/map/deforestation). The Real-Time Deforestation Detection System (DETER) of the National Institute for Space Research (INPE) uses images from the WFI/CBERS-4 and AWiFS/IRS sensors that cover the Cerrado every five days and make it possible to detect deforestation polygons with an area larger than 0.03 km^2^ [6]. The high availability of the images used by DETER makes the system an ideal tool for quickly informing enforcement agencies of new deforestation.

### Statistical analysis

Statistical analysis was performed on R software version 4.0.3 [26]. For each state, the Poisson generalized linear model (PGLM) was adjusted between dengue fever cases per 100,000 inhabitants (dependent variable) as function of loss of vegetation (km²) and year. PGLM is suitable as it can include non-continuous variables like data count of dengue fever cases, account for nonlinear relationships and handle non-Gaussian error distributions [27]. The significance of the coefficients obtained was verified by the by Wald-test. We checked the models for temporal dependence applied Durbin-Watson test for autocorrelation values by “DHARma” package. This R package uses a simulationbased approach to create readily interpretable scaled residuals from fitted PGLM for each state [28]. Boxplot graphs were prepared for data on cases of dengue fever and loss of vegetation associated with Brazilian states located in the Cerrado biome on an annual scale [26]. The statistical analysis was based on the Mann-Kendall test [29], seeking to identify significant trends in cases of dengue fever and loss of vegetation using data of S1 Table. This procedure considers the hypothetical stability of successive and independent values with the maintenance of the same probability distribution. The variables were subjected to the non-parametric Pettitt test [30, 31], which identifies years with an abrupt change in the time series. Based on the Pettitt and Mann-Kendal test, maps were drawn for each Brazilian state for years in which an abrupt change in the time series for loss of native vegetation occurred [27, 32–34].

Cluster analysis was applied to identify states with a homogeneous distribution of loss of native vegetation and dengue fever over time [35]. For this purpose, a joint data analysis (paired data) on the loss of native vegetation and the incidence of dengue cases per 100,000 inhabitants were separated by states to identify possible clusters. The Euclidean distance was considered a measure of dissimilarity on the calculation of distances between states [36, 37].

Time series are analyzed to understand the past and predict the future, allowing managers or policymakers to make decisions. A time-series analysis quantifies the main characteristics of a data set and its random variation. Combined with enhanced computing power, this time series analysis feature has made time series methods widely applicable in government, industry, and commerce [38].

For this study, the R package [26] called *forecast* [39, 40] was used to perform the analysis of time series and predict future data as an automatic ARIMA model [41, 42]. A function was defined separately for the cases of dengue and loss of native vegetation for each Brazilian state, generating a function for each variable, and its parameters were automatically adjusted using the minimization of the AIC [43] to adjust the variability curve of the original data with the trend, seasonal, cyclical and irregular components. Furthermore, the same parameters were used to predict the next steps in time.

### Model validation for dengue fever cases and loss of vegetation in Cerrado biome

To evaluate and choose the best model, there are some fundamental procedures to identify the orders of the ARIMA models. Akaike information criterion (AIC) [43], Bayesian information criterion (BIC) [44], and other measures such as ME (Mean Error), RMSE (Root Mean Square), MAE (Mean Absolute Error), MPE (Mean Percentage Error), MAPE (Mean Absolute Percentage Error), and ACF1 (the first order partial autocorrelation coefficient) were estimated (S2 and S3 Tables) for each state and each variable evaluated (dengue fever cases and loss of native vegetation in the Cerrado).

## Results

### Modeling of the dengue fever cases as function of loss of vegetation and year

The Durbin-Watson test showed that the autocorrelation of the residuals of the Poisson generalized linear model (PGLM) adjusted between dengue cases per 100,000 inhabitants (dependent variable) as function of deforestation (km²) and year for each state of the Brazilian Cerrado are equal to zero for all states of the Brazilian Cerrado (Table 1). All adjusted PGLM coefficients were significant by the Wald-test. This demonstrates that they are statistically different from zero.

**Table 1:** P-value of the Durbin-Watson (DW) test for the autocorrelation analysis and Poisson generalized linear model coefficients adjusted between dengue cases per 100,000 inhabitants (dependent variable) as function of deforestation (km²) and year for each state of the Brazilian Cerrado.

Brazilian states	DW	β_0_	Deforestation	Year
BA	0.4026	69.87*	-0.00027*	-0.0315*
DF	0.5240	-280.90*	-0.00425*	0.1424*
GO	0.1200	-265.10*	-0.00142*	0.1357*
MA	0.8281	62.58*	-0.00028*	0.0285*
MG	0.6907	-271.70*	-0.00004*	0.1383*
MS	0.6384	-59.98*	-0.00019*	0.0334*
MT	0.5168	121.30*	-0.00041*	-0.0566*
PI	0.4619	44.39*	-0.00020*	-0.0193*
SP	0.4735	-225.40*	-0.00139*	0.1151*
TO	0.4715	62.01*	-0.00055*	-0.0271*

*: Significant at 1% probability by Wald-test; Brazilian states: BA—Bahia, DF–Distrito Federal, GO—Goiás, MA—Maranhão, MG—Minas Gerais, MS—Mato Grosso do Sul, MT—Mato Grosso, PI—Piauí, SP—São Paulo and TO—Tocantins.

### Descriptive statistics and cluster analysis for dengue fever cases and loss of vegetation

**Fig 2:**
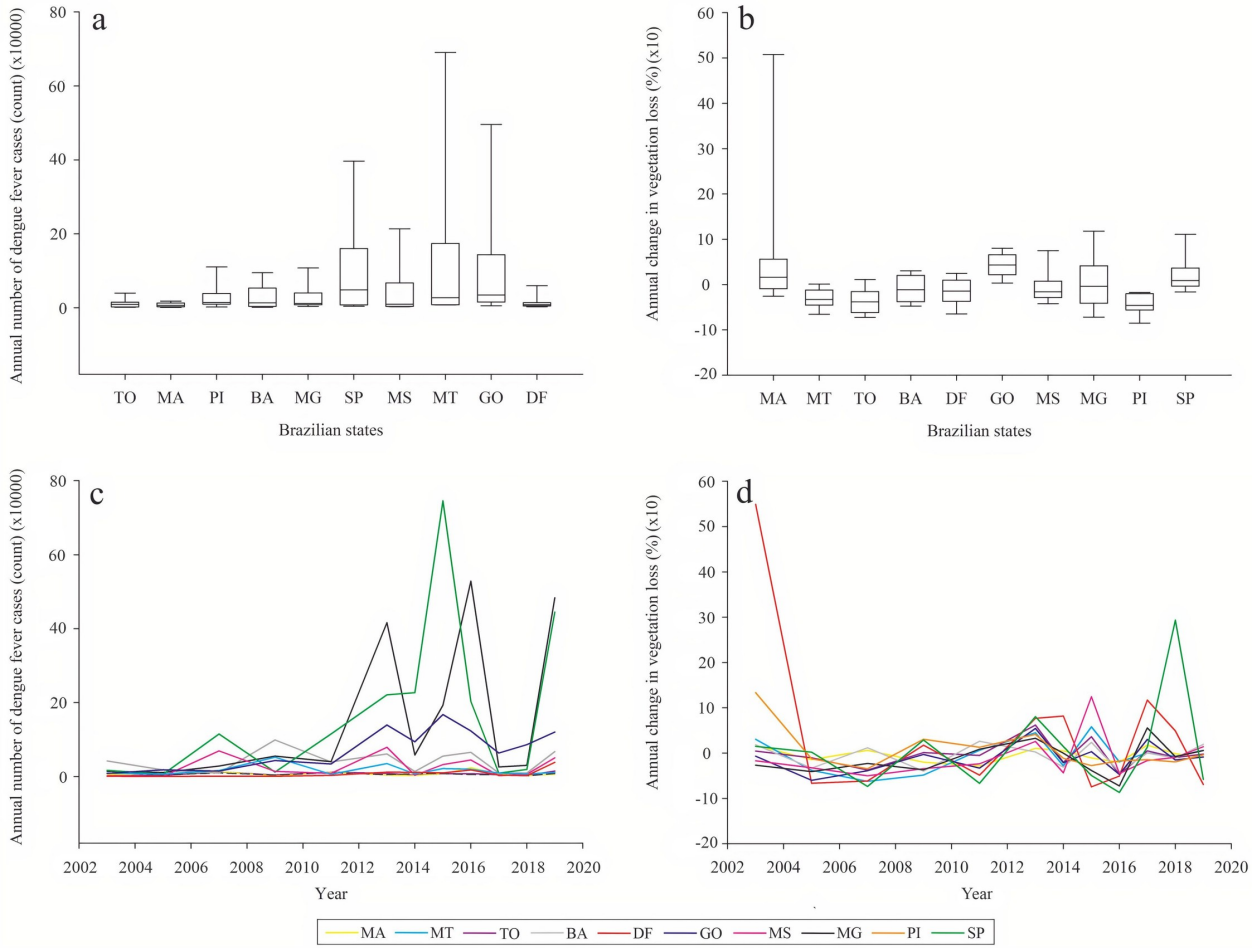
Boxplot applied to the absolute values of the annual cases of dengue fever and loss of vegetation (km²) and temporal evolution of these variables between 2001 and 2019. a: boxplot of annual dengue cases, b: boxplot of loss of native vegetation in the Cerrado, c: temporal evolution of annual dengue cases, d: temporal evolution of loss of native vegetation in the Cerrado.

Between 2001 and 2019, 7,950,624 dengue cases were recorded across the Brazilian Cerrado, and 281,833.32 km^2^ of native area were deforested in the same period (Fig 2). The state of Mato Grosso (MT) had the highest variability of annual number of dengue fever cases, while the state of Maranhão (MA) had the highest variability of annual change in vegetation loss.

**Fig 3:**
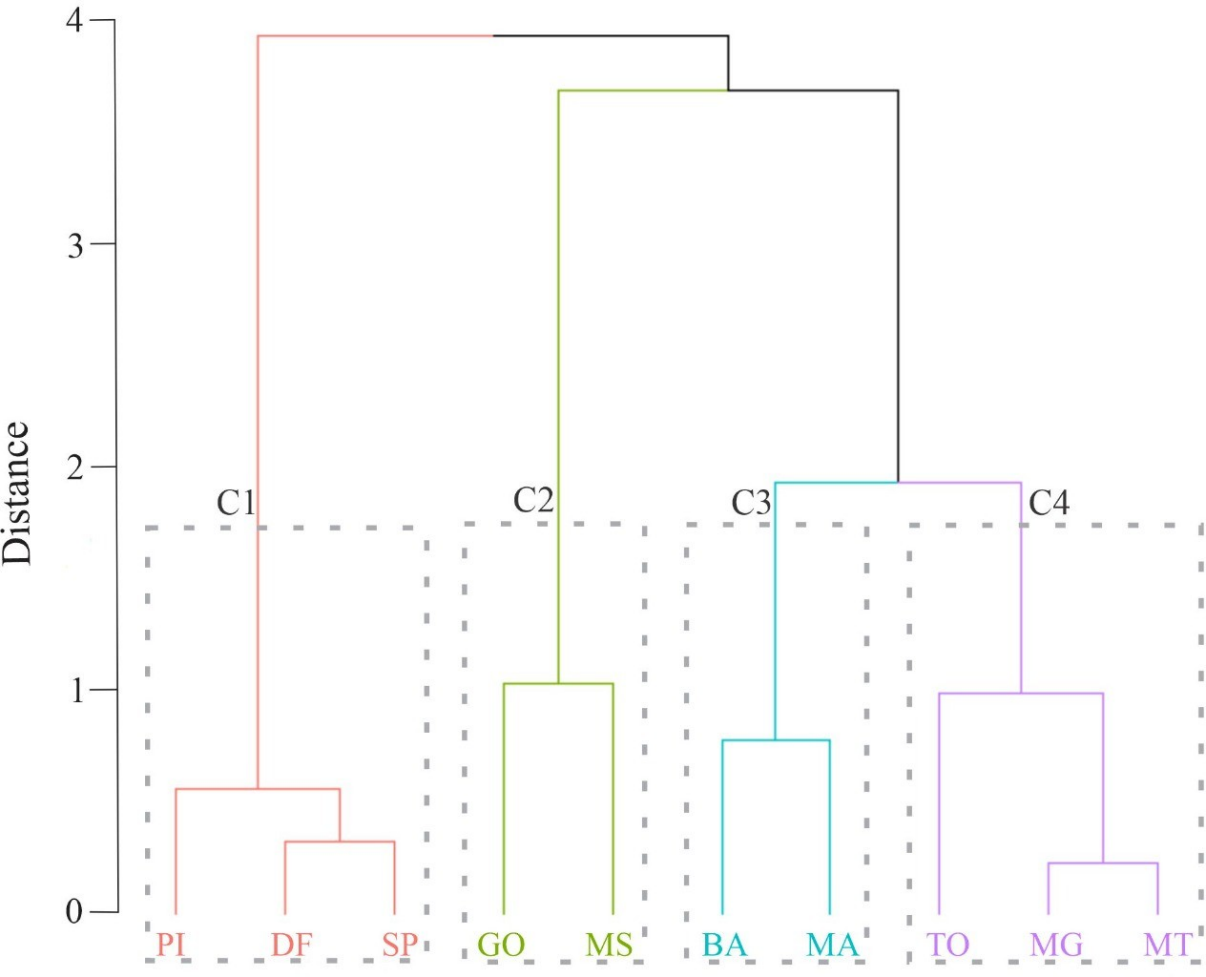
Cluster analysis applied to the Cerrado biome using annual dengue fever cases and loss of vegetation. C1: Cluster I, C2: Cluster II, C3: Cluster III and C4 Cluster IV.

Cluster analysis revealed four clusters (Fig 3). Cluster I, composed of the states of Piauí, Distrito Federal and São Paulo, were grouped because they presented low values for the incidence of dengue cases per 100,000 inhabitants and for deforestation. Cluster II, on the other hand, includes the states of Goiás and Mato Grosso do Sul, grouped together because they present high incidence of dengue cases. The states of Bahia and Maranhão formed Cluster III, for presenting low incidence of dengue cases and moderate deforestation, compared to the other states. Cluster IV, formed by the states of Tocantins, Minas Gerais and Mato Grosso, was clustered because it had a moderate incidence of dengue cases and high values for loss of native vegetation.

### Temporal patterns for annual cases of dengue fever and loss of vegetation

**Fig 4:**
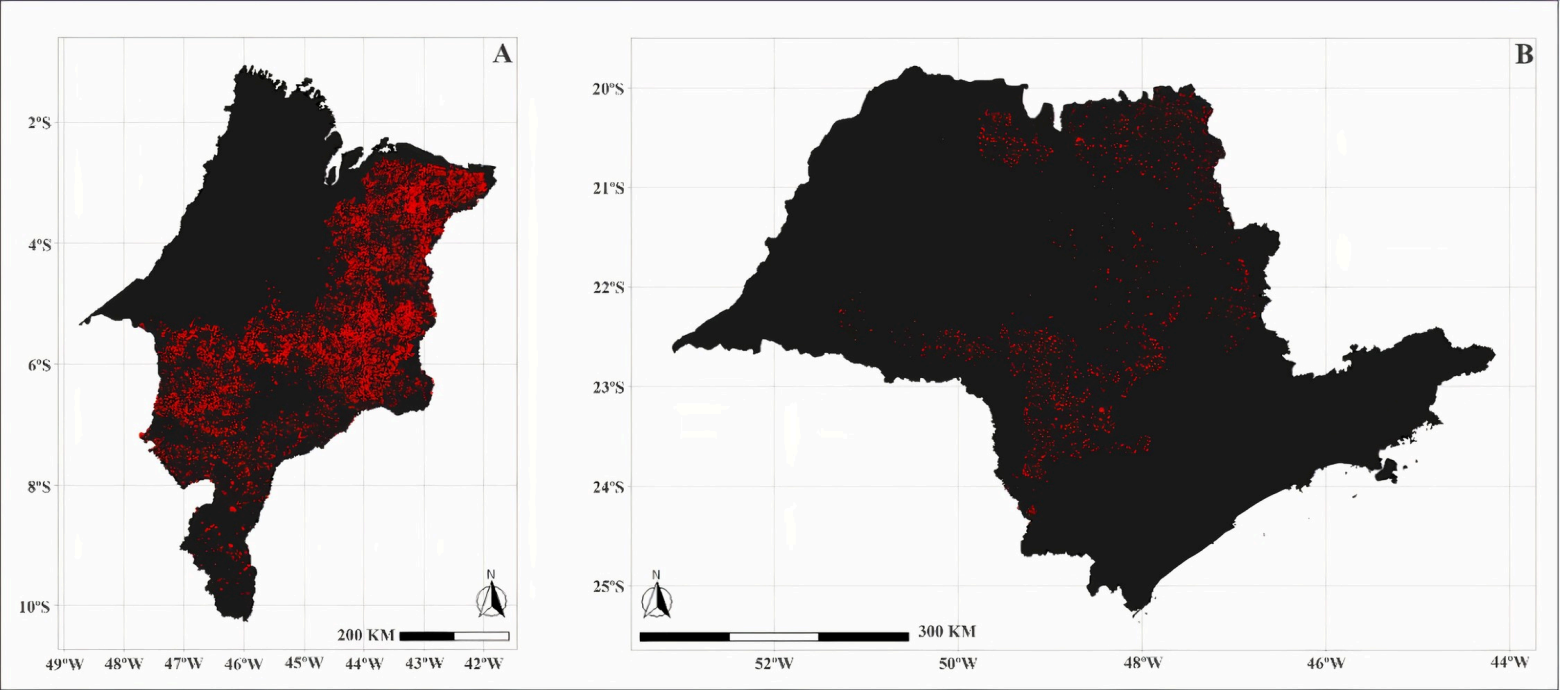
Loss of native vegetation (km²) in the states of Maranhão (A) and São Paulo (B) in 2009 and 2010, respectively. The red region corresponds to the area deforested in the year indicated by the Pettitt and Mann-Kendal test.

**Fig 5:**
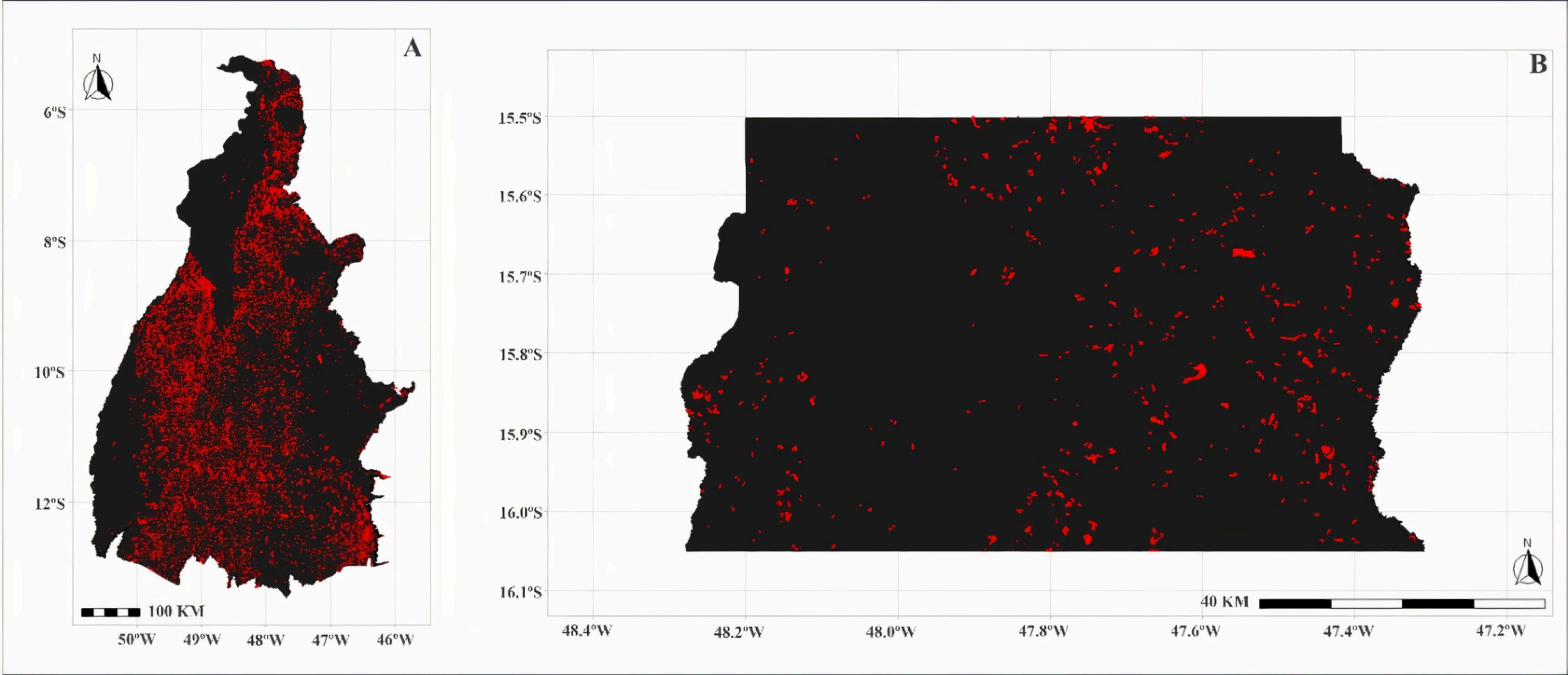
Loss of native vegetation (km²) in the states of Tocantins (A) and Federal District (B) in 2006. The red region corresponds to the area deforested in the year indicated by the Pettitt and Mann-Kendal test.

**Fig 6:**
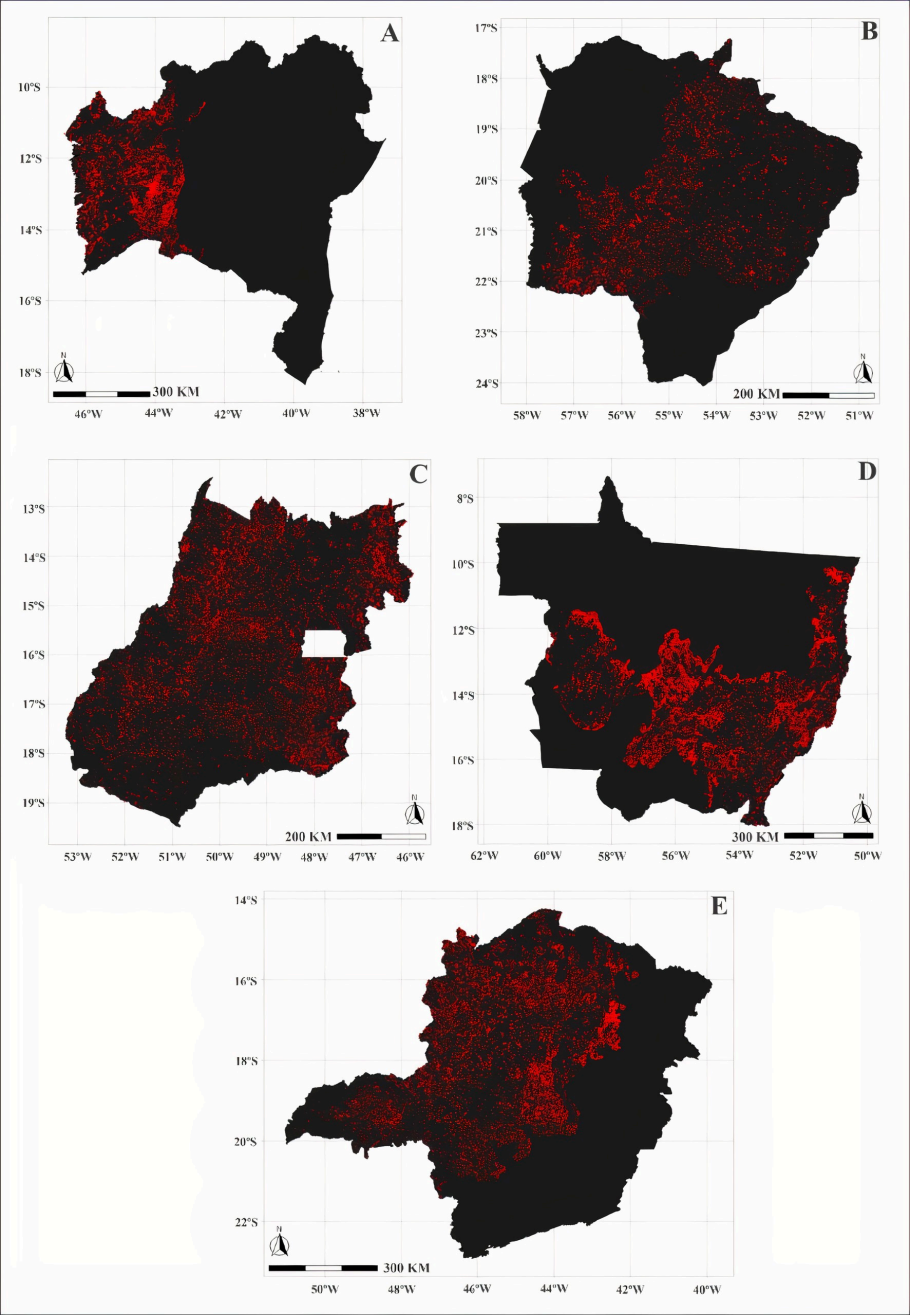
Loss of native vegetation (km²) in the states of Bahia (A), Mato Grosso do Sul (B), Goiás (C), Mato Grosso (D) and Minas Gerais (E) in 2008. The red region corresponds to the area deforested in the year indicated by the Pettitt and Mann-Kendal test.

Trend analysis was performed for data on annual dengue fever cases and native loss of vegetation (Table 1). The Mann-Kendall test highlighted a significant trend of increasing dengue cases for the Brazilian states Federal District (DF), Goiás (GO), and Minas Gerais (MG). Pettitt’s test identified inflection points in the patterns of dengue fever cases for the Federal District (DF), Goiás (GO), and MG in 2009, 2007, and 2007, respectively. As for the loss of vegetation in the Cerrado, the Mann-Kendall test highlighted a significant upward trend for all Brazilian states except the Piauí (PI). Pettitt’s test identified inflection points in vegetation loss patterns for São Paulo (SP) in 2010, MA in 2009, Bahia (BA), GO, MG, Mato Grosso do Sul (MS), and Mato Grosso (MT) in 2008, DF and Tocantins (TO) in 2006 (Table 2 and Figs 4–6).

**Table 2:** P-values of the Mann-Kendall (MK) and Pettitt trend tests applied to cases of annual dengue fever infection and loss of native vegetation and years identified by the Pettitt test as a likely point of change in the time series for each Brazilian Cerrado state.

Brazilian states	Loss of vegetation			Dengue fever		
	MK	Pettitt	Year	MK	Pettitt	Year
BA	<0.001	0.003	2008	0.330	0.460	-
DF	<0.001	0.021	2006	0.004	0.006	2009
GO	<0.001	0.003	2008	0.001	0.006	2007
MA	<0.001	0.002	2009	0.670	0.999	-
MG	<0.001	0.003	2008	0.017	0.043	2007
MS	<0.001	0.003	2008	0.260	0.460	-
MT	0.004	0.003	2008	0.180	0.210	-
PI	0.399	0.400	-	0.780	0.760	-
SP	<0.001	0.002	2010	0.051	0.100	-
TO	<0.001	0.043	2006	0.210	0.820	-

Brazilian states: BA—Bahia, DF–Distrito Federal, GO—Goiás, MA—Maranhão, MG—Minas Gerais, MS—Mato Grosso do Sul, MT—Mato Grosso, PI—Piauí, SP—São Paulo and TO—Tocantins.

### ARIMA model for the cases of dengue and loss of native vegetation

**Fig 7:**
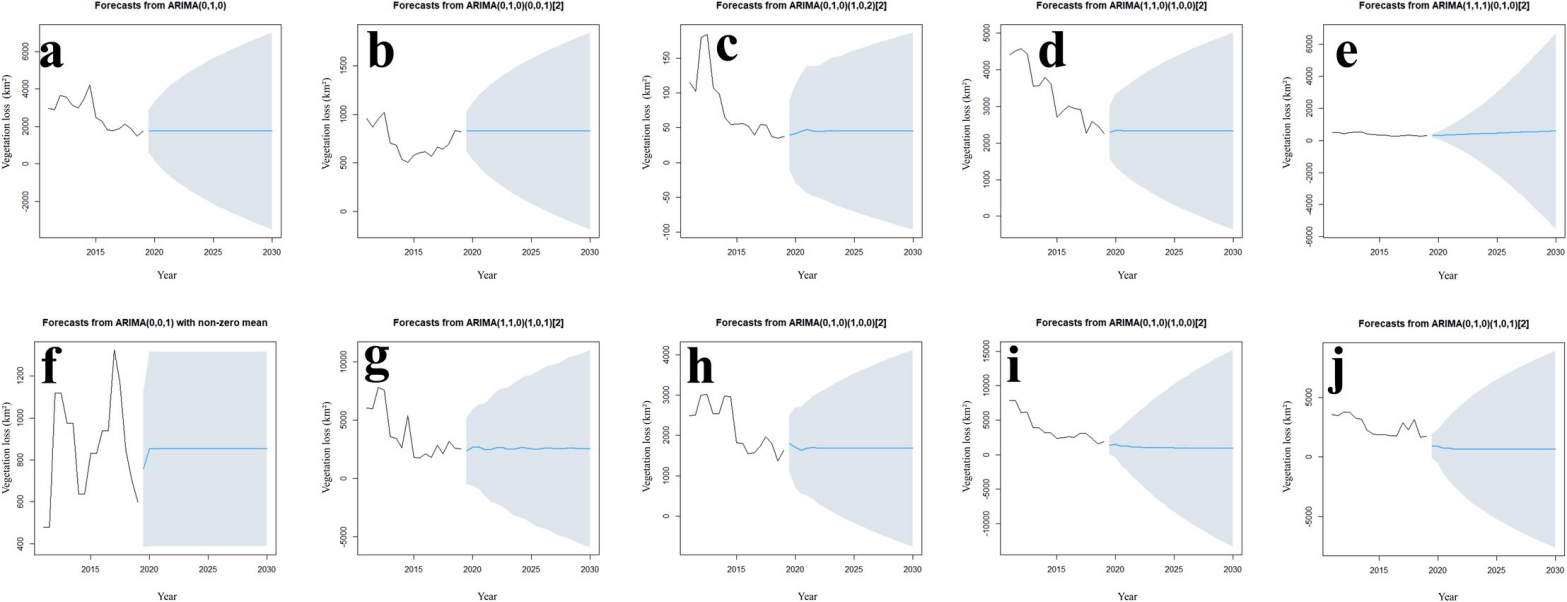
ARIMA models for loss of vegetation for each Brazilian Cerrado state. The solid black line is the original data, the predicted blue line starts after 19 years and the gray area is the 95% confidence level. a: Bahia (BA), b: Distrito Federal (DF), c: Goiás (GO), d: Maranhão (MA), e: Minas Gerais (MG), f: Mato Grosso do Sul (MS), g: Mato Grosso (MT), h: Piauí (PI), i: São Paulo (SP), and j: Tocantins (TO).

Forecasts suggest that the loss of vegetation in the Cerrado will experience stability in the next decade, but before that happens, in some states there will be an increase (Piauí, Goiás and Mato Grosso) and others a reduction (Tocantins, Bahia, Federal District, Minas Gerais and Mato Grosso do Sul) (Fig 7).

**Fig 8:**
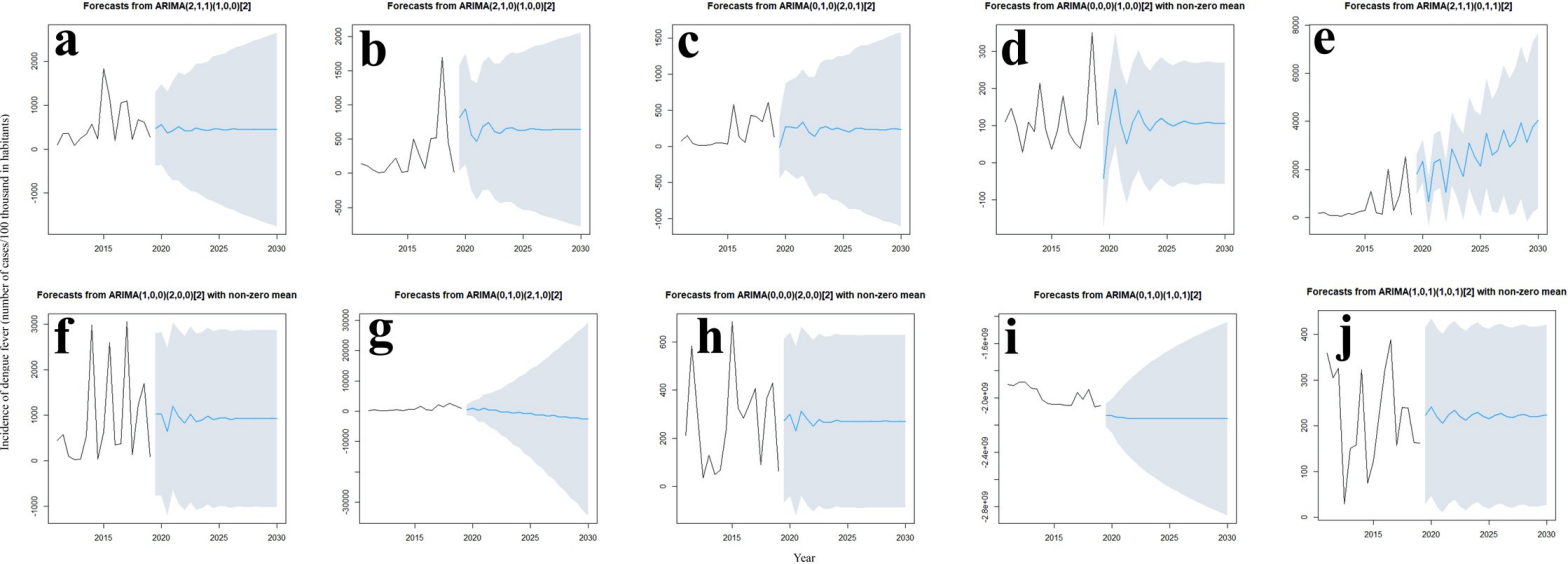
ARIMA models for dengue fever cases for each Brazilian Cerrado state. The solid black line is the original data, the predicted blue line starts after 19 years and the gray area is the 95% confidence level. a: Bahia (BA), b: Distrito Federal (DF), c: Goiás (GO), d: Maranhão (MA), e: Minas Gerais (MG), f: Mato Grosso do Sul (MS), g: Mato Grosso (MT), h: Piauí (PI), i: São Paulo (SP), and j: Tocantins (TO).

For dengue fever cases, forecasts indicate that slight variations will occur for Bahia, Federal District, Goiás, Maranhão, Mato Grosso do Sul, Mato Grosso, Piauí, São Paulo, and Tocantins states, however, the trend is to stabilize and remain constant (Fig 8). The forecast for the state of Minas Gerais indicates increases in the incidence of dengue cases per 100,000 inhabitants between the period considered.

## Discussion

This research found a significant relationship between loss of native vegetation and years with dengue cases in the Cerrado biome. The adjusted regression coefficients for deforestation were negative and statistically different from zero for all Brazilian Cerrado states. It is also important to highlight that the residuals of the fitted models do not show spatial dependence.

In this study, the loss of native vegetation and the increase in dengue cases measured between 2001 and 2019 are prevalent in Mato Grosso, São Paulo, and Mato Grosso do Sul. These states showed a significant trend of vegetation loss for the evaluated time series, with an inflection point in the years 2008, 2010 and 2008, respectively. However, several similar studies [12, 15, 20, 57] point out the damage caused by deforestation in the communities’ health that lives close to tropical forests is significant. Mainly the improvement of environmental conditions for the mosquito vectors, since the loss of vegetation near urban centers causes an increase in air temperature and food availability.

In some states, such as Minas Gerais, which had a low rate of deforestation in the studied period, there was a higher intensification in previous times, mainly due to its occupation dating from the 16th century. There were many cases of dengue fever, being identified in this study a trend of the high incidence of cases in the next ten years, the highest observed in this study. A similar situation occurs in São Paulo, but with a tendency to stabilize around 300 thousand annual cases from 2025 to 2030, but this state has become more anthropogenic more quickly than Minas Gerais.

As seen in the cluster analysis, there was clustering in different groups, which reinforces the idea that there is a need for public health and environmental policies directed to each region because it is a vast geographical region. Such information collaborates with the development of public policies and government planning strategies, contributing to the environmental conservation and reducing dengue cases in populations residing in that biome. According to the cluster analysis, it is evident that each region within the Cerrado has particularities, and the Federal Government should institute environmental and sanitary public policies directed to each region. In addition, these results associated with future modeling performed in this research allows for personalized and joint interventions by state governments to reduce the dengue incidence and deforestation rate.

The central portion of Brazil started to be occupied in the 16th century, but it experienced a high anthropization and development of agriculture in that region in the 1960s and 1970s [5], for several reasons, in which the most important was the climate and the soil [45]. As a consequence, there was a higher intensification of deforestation [46] and, with that, it caused the deregulation of ecological systems [47], mainly concerning *Aedes aegypti* L. predators both on a regional and global scale [48]. Unlike the *Aedes aegypti*, which has a cosmopolitan characteristic, its main predators such as the mosquito *Toxorhynchites theobaldi* [49], dragonflies [50, 51], and several species of fish [52–54], need specific conditions for reproduction and development. In this sense, the loss of native vegetation areas, far away or close to populated areas, would eventually reduce environments suitable for these species [55], reducing and often isolating the populations of predators of the *Aedes aegypti*, making its control dependent mainly on public policies of the Health System.

Our results indicate significant tendency in vegetation loss, with the exception of the state of Piauí. However, it is worth noting that in 2020 (year that was not included in our time series) there were significant fires in all biomes, with the Cerrado being one of the most affected, which may influence new predictions of future scenarios. In 2020, the state of Mato Grosso lost a large area of native vegetation [6], mainly due to the negligence of the federal government in the face of the fires that occurred there probably associated with the opening of new agricultural areas. These facts can promote an increase in infectious diseases in the populations that live there [14, 15, 48]. It is also necessary to associate these events with socioeconomic indices such as population growth, population income, human development, unemployment in this region and that there is a partition of the biome for the development of public policies and their more effective applicability, especially in the states of Minas Gerais, São Paulo, and Mato Grosso.

MacDonald and Mordecai [20] used techniques such as regionalization of areas in Amazon to more effectively identify cases of malaria and deforestation in this forest, which made it clear the importance that native areas have for local public health. The Amazon rainforest and the Cerrado are centers of global biodiversity, presenting one of the highest rates of endemism in the world’s biomes, and their loss would not only affect Brazil.

The acceleration of the loss of native vegetation in the Cerrado is associated with human activities, especially the expansion of the agricultural area, highlighting the concern of future generations with the loss of biodiversity, sustainable development, and public health. As the Cerrado occupies the second largest territorial portion of Brazil, it was found in another study that this biome had the second-highest rate of vegetation loss [56], thus revealing a need for greater concern in this biome.

The Federal Government’s negligence with environmental and health public policies is evident. This is supported by the forest fires that occurred in 2020 [57], and the position occupied by Brazil in the world ranking of Coronavirus disease (COVID-19), 3rd place with more than 18 million confirmed cases and 2nd place with more than 500 thousand deaths [58]. And yet, regarding dengue fever, there has been an increase in cases of infection between the years 2018 and 2019.

If more effective public policies are not implemented, it is expected that the populations living in the Brazilian Cerrado will experience an increase in cases of dengue fever infection and deforestation, especially in the southeast (Minas Gerais and São Paulo) and northwest (Mato Grosso) of this biome. Studies in tropical regions have found a significant increase in infectious diseases [11, 12, 18, 19, 59, 60], a common factor observed among these studies is the more favorable environment for the vectors, mainly by the decrease of natural predators (for example, dragonflies, fry, and fungi), increased air temperature, and increased availability of food (algae), the latter two being accelerators of the vectors’ life cycle. These researchers reported that even under more pessimistic scenarios, the potential to reduce cases of infectious diseases in tropical regions will only occur with an extreme decrease in deforestation and implementation of public policies seeking at controlling, inspecting, and conserving these areas, in addition to improving the living conditions of the populations inhabiting the Cerrado.

### Limitations and implications of the study

Some limitations of our study should be taken into consideration. First, we performed only time series analysis without considering the factors that affected the occurrence of dengue and deforestation, such as separation of incidence of dengue cases in urban and rural populations, climatic and socioeconomic variations (e.g. social inequality, per capita income, Human Development Index, GINI Index, GDP). Second, since the National System of Notifiable Diseases (SINAN) was created in 1993, and the Ministerial Ordinance that regulates and creates the national list of compulsorily notifiable diseases was published in December 1999, we could only obtain data from 2001 to 2019. It is also worth noting the possibility of underreporting in reported cases of dengue, due to several factors, mainly due to asymptomatic cases and the failure to seek medical care. More data can improve the effectiveness of the PGLM adjusted for each state of Brazilian Cerrado.

Third, this study focused only on the Cerrado biome. Whether these models are suitable for other epidemic locations or other infectious diseases requires further and robust study, as well as the use of other model prediction methods such as Ecological Niches Modeling. It is known that climatic variables (e.g. high temperatures and rainfall) favor the ecology of the vector (*A*. *aegypti)* and the spread of the disease [21]. However, we believe that these variables can be incorporated in future studies.

It is also worth noting the existence of underreporting dengue cases in the country, either mainly due to the distance of rural populations to health units, or the characteristics of these populations, and even to the increase in other diseases as has been happening with Coronavirus disease (COVID-19), in which the health system of several states has collapsed, making arboviroses such as dengue less evident.

These facts added to what is available in the literature in which the loss of vegetation is directly linked to the increase of other tropical infectious diseases makes clear the need for further studies addressing this issue. Thus, the following recommendations are derived from this study: (i) add socioeconomic variables (for example, Human Development Index, Gini Index, Urbanization rate, GDP, per capita income, access to health facilities, investment in public health, population growth after deforestation) and environmental variables (e.g. temperature, rainfall, relative humidity, land cover and land use after vegetation removal) to the predictive models; (ii) test different prediction methods in an attempt to validate the most efficient and adequate model, and (iii) test these models for other arboviroses or other tropical diseases.

## Conclusions

The Cerrado biome is responsible for a large part of Brazil’s agricultural production and concentrates a vast population. Over decades, this biome has suffered from the loss of vegetation and various ecosystem services. Deforestation in the Cerrado affects the environment and the resident populations in several ways. This study finds a relationship between deforestation and an increase in dengue cases. However, this study shows worrying forecasts for dengue cases in Minas Gerais, São Paulo, and Mato Grosso. To reduce the incidence of dengue cases in the Cerrado, Brazil will need to implement public health, environmental and social policies, requiring a joint effort by all spheres of society.

## Supporting information

S1 Table Data used to perform the trend test and generalized linear models for the variables dengue fever cases (number of cases per 100 thousand in habitants) and loss of native vegetation (km²) in the Cerrado in Brazilian states. Brazilian states: BA—Bahia, DF–Distrito Federal, GO—Goiás, MA—Maranhão, MG—Minas Gerais, MS—Mato Grosso do Sul, MT—Mato Grosso, PI—Piauí, SP—São Paulo and TO—Tocantins.
(DOCX)

S2 Table Statistical parameters obtained for the ARIMA model adjusted for dengue fever cases in Brazilian states. AIC: Akaike information criterion, BIC: Bayesian information criterion, ME: Mean Error, RMSE: Root Mean Square, MAE: Mean Absolute Error, MPE: Mean Percentage Error, MAPE: Mean Absolute Percentage Error, ACF1: first-order partial autocorrelation coefficient. Brazilian states: BA—Bahia, DF–Distrito Federal, GO—Goiás, MA—Maranhão, MG—Minas Gerais, MS—Mato Grosso do Sul, MT—Mato Grosso, PI—Piauí, SP—São Paulo and TO—Tocantins.
(DOCX)

S3 Table Statistical parameters obtained for the ARIMA model adjusted for loss of native vegetation in the Cerrado in Brazilian states. AIC: Akaike information criterion, BIC: Bayesian information criterion, ME: Mean Error, RMSE: Root Mean Square, MAE: Mean Absolute Error, MPE: Mean Percentage Error, MAPE: Mean Absolute Percentage Error, ACF1: first-order partial autocorrelation coefficient. Brazilian states: BA—Bahia, DF–Distrito Federal, GO—Goiás, MA—Maranhão, MG—Minas Gerais, MS—Mato Grosso do Sul, MT—Mato Grosso, PI—Piauí, SP—São Paulo and TO—Tocantins.
(DOCX)
