# Supplementary material for: The fewer, the better fare: Can the loss of vegetation in the Cerrado drive the increase in dengue fever cases infection?
Source: PLoS One. 2022 Jan 13;17(1):e0262473. doi: 10.1371/journal.pone.0262473 (PMC8757950; doi:10.1371/journal.pone.0262473)
Supplement: S1 Table — Brazilian states: BA—Bahia, DF–Distrito Federal, GO—Goiás, MA—Maranhão, MG—Minas Gerais, MS—Mato Grosso do Sul, MT—Mato Grosso, PI—Piauí, SP—São Paulo and TO—Tocantins. (DOCX) [file pone.0262473.s001.docx]

Table S1. Data used to perform the trend test and generalized linear models for the variables dengue fever cases (number of cases per 100 thousand in habitants) and loss of native vegetation (km²) in the Cerrado in Brazilian states.

| STATE | YEAR | DENGUE | DEFORESTATION |
| --- | --- | --- | --- |
| BA | 2001 | 212.6 | 4400.83 |
| BA | 2002 | 582.4 | 4520.1 |
| BA | 2003 | 315.3 | 4568.3 |
| BA | 2004 | 34.4 | 4422.5 |
| BA | 2005 | 129.5 | 3537.6 |
| BA | 2006 | 49 | 3571.2 |
| BA | 2007 | 67.7 | 3783 |
| BA | 2008 | 238.2 | 3606.5 |
| BA | 2009 | 683.4 | 2700.2 |
| BA | 2010 | 324.1 | 2877.3 |
| BA | 2011 | 282.6 | 3008.4 |
| BA | 2012 | 342 | 2937.3 |
| BA | 2013 | 406.2 | 2906.8 |
| BA | 2014 | 91.4 | 2270.7 |
| BA | 2015 | 365 | 2586.3 |
| BA | 2016 | 429.8 | 2456.5 |
| BA | 2017 | 63.2 | 2258.6 |
| BA | 2018 | 64.6 | 2364.1 |
| BA | 2019 | 453.7 | 2392.7 |
| DF | 2001 | 75.4 | 115.63 |
| DF | 2002 | 147.5 | 102.5 |
| DF | 2003 | 40.6 | 180.8 |
| DF | 2004 | 11.7 | 184.4 |
| DF | 2005 | 16.8 | 107.8 |
| DF | 2006 | 22.1 | 98.5 |
| DF | 2007 | 47.5 | 65.1 |
| DF | 2008 | 45.9 | 54.7 |
| DF | 2009 | 34.8 | 55.1 |
| DF | 2010 | 584.3 | 56.2 |
| DF | 2011 | 129.4 | 52.9 |
| DF | 2012 | 54.2 | 39.2 |
| DF | 2013 | 428.4 | 55 |
| DF | 2014 | 408.7 | 54.1 |
| DF | 2015 | 345.5 | 37.1 |
| DF | 2016 | 611.9 | 35.4 |
| DF | 2017 | 129.8 | 37.1 |
| DF | 2018 | 79.1 | 44.2 |
| DF | 2019 | 1272.7 | 37.1 |
| GO | 2001 | 210.5 | 957.91 |
| GO | 2002 | 421.6 | 871.1 |
| GO | 2003 | 161.5 | 953.3 |
| GO | 2004 | 108.1 | 1019.8 |
| GO | 2005 | 335.3 | 703.8 |
| GO | 2006 | 424.4 | 680.5 |
| GO | 2007 | 166.5 | 539.4 |
| GO | 2008 | 609.4 | 505.2 |
| GO | 2009 | 646.4 | 581.9 |
| GO | 2010 | 1700.1 | 606.4 |
| GO | 2011 | 565.6 | 617.2 |
| GO | 2012 | 398.3 | 567.7 |
| GO | 2013 | 2165.9 | 661.5 |
| GO | 2014 | 1439.9 | 643 |
| GO | 2015 | 2566.6 | 698.7 |
| GO | 2016 | 1863.6 | 832.3 |
| GO | 2017 | 936.7 | 822.5 |
| GO | 2018 | 1246.9 | 921.2 |
| GO | 2019 | 1714.3 | 936.4 |
| MA | 2001 | 109.6 | 2950.02 |
| MA | 2002 | 146.3 | 2906.1 |
| MA | 2003 | 99.8 | 3661.6 |
| MA | 2004 | 27.4 | 3588.8 |
| MA | 2005 | 108.3 | 3157.6 |
| MA | 2006 | 83.8 | 2994.1 |
| MA | 2007 | 214.1 | 3434 |
| MA | 2008 | 88.5 | 4211.3 |
| MA | 2009 | 36 | 2474 |
| MA | 2010 | 87.9 | 2290.5 |
| MA | 2011 | 179.1 | 1803.5 |
| MA | 2012 | 79.3 | 1765.2 |
| MA | 2013 | 52.8 | 1884.8 |
| MA | 2014 | 38.7 | 2119.6 |
| MA | 2015 | 115.9 | 1871.9 |
| MA | 2016 | 350 | 1475.1 |
| MA | 2017 | 101.9 | 1752.5 |
| MA | 2018 | 30.1 | 1656.8 |
| MA | 2019 | 79.7 | 1581.8 |
| MG | 2001 | 178.3 | 7873.53 |
| MG | 2002 | 210.1 | 7850.9 |
| MG | 2003 | 77.3 | 6066.9 |
| MG | 2004 | 73.2 | 6202 |
| MG | 2005 | 58.8 | 3942.4 |
| MG | 2006 | 154.4 | 3882.6 |
| MG | 2007 | 143.9 | 3195.8 |
| MG | 2008 | 248.8 | 3197.8 |
| MG | 2009 | 283.1 | 2416.6 |
| MG | 2010 | 1094.8 | 2456.7 |
| MG | 2011 | 205.9 | 2573.1 |
| MG | 2012 | 148.4 | 2496.5 |
| MG | 2013 | 2021.3 | 3134.3 |
| MG | 2014 | 280.6 | 3137.4 |
| MG | 2015 | 929.8 | 2393.7 |
| MG | 2016 | 2532.2 | 1604 |
| MG | 2017 | 122.9 | 1849.3 |
| MG | 2018 | 144.1 | 1923.4 |
| MG | 2019 | 2284.2 | 1978.9 |
| MS | 2001 | 447.2 | 2489.41 |
| MS | 2002 | 568.8 | 2496.9 |
| MS | 2003 | 97.2 | 3005.2 |
| MS | 2004 | 14.6 | 3011.8 |
| MS | 2005 | 28.2 | 2542.7 |
| MS | 2006 | 538.6 | 2535.2 |
| MS | 2007 | 2976 | 2975.5 |
| MS | 2008 | 32.8 | 2966.2 |
| MS | 2009 | 625.6 | 1815.3 |
| MS | 2010 | 2593.6 | 1807.9 |
| MS | 2011 | 347.5 | 1548.9 |
| MS | 2012 | 367.3 | 1565.4 |
| MS | 2013 | 3051.8 | 1730.8 |
| MS | 2014 | 130.7 | 1970.5 |
| MS | 2015 | 1230.7 | 1798.6 |
| MS | 2016 | 1690.3 | 1372.6 |
| MS | 2017 | 84.6 | 1635.5 |
| MS | 2018 | 225.5 | 1640.6 |
| MS | 2019 | 1820.5 | 1489.7 |
| MT | 2001 | 101.4 | 6059.2 |
| MT | 2002 | 356.9 | 6004.6 |
| MT | 2003 | 357.1 | 7805.9 |
| MT | 2004 | 88.9 | 7592.9 |
| MT | 2005 | 245.1 | 3581.7 |
| MT | 2006 | 348.8 | 3449.6 |
| MT | 2007 | 565.4 | 2638.3 |
| MT | 2008 | 227.8 | 5412.7 |
| MT | 2009 | 1839.2 | 1820.3 |
| MT | 2010 | 1167.8 | 1774.7 |
| MT | 2011 | 202.5 | 2093.7 |
| MT | 2012 | 1054.7 | 1822.5 |
| MT | 2013 | 1105.9 | 2863.8 |
| MT | 2014 | 222.1 | 2115.1 |
| MT | 2015 | 669.4 | 3190.9 |
| MT | 2016 | 610.6 | 2566.1 |
| MT | 2017 | 275.9 | 2557.7 |
| MT | 2018 | 209.5 | 2437.6 |
| MT | 2019 | 303.7 | 2817.2 |
| PI | 2001 | 359 | 479.8 |
| PI | 2002 | 305.1 | 479.8 |
| PI | 2003 | 325.1 | 1119.2 |
| PI | 2004 | 29.6 | 1119.2 |
| PI | 2005 | 150.7 | 976.2 |
| PI | 2006 | 158.5 | 976.2 |
| PI | 2007 | 321.8 | 637.6 |
| PI | 2008 | 75.1 | 637.6 |
| PI | 2009 | 123.7 | 832.2 |
| PI | 2010 | 228.9 | 832.2 |
| PI | 2011 | 322.6 | 939.1 |
| PI | 2012 | 387.7 | 939.1 |
| PI | 2013 | 156.6 | 1325.9 |
| PI | 2014 | 239.7 | 1171.7 |
| PI | 2015 | 239.3 | 847 |
| PI | 2016 | 163.6 | 700.8 |
| PI | 2017 | 162.8 | 599.2 |
| PI | 2018 | 57.5 | 482.6 |
| PI | 2019 | 243 | 463 |
| SP | 2001 | 137.3 | 502.5 |
| SP | 2002 | 110.4 | 496 |
| SP | 2003 | 52.6 | 444.3 |
| SP | 2004 | 7.8 | 502.1 |
| SP | 2005 | 14.3 | 537.2 |
| SP | 2006 | 130.8 | 531.6 |
| SP | 2007 | 221.6 | 386 |
| SP | 2008 | 17.9 | 384.8 |
| SP | 2009 | 21.8 | 346 |
| SP | 2010 | 503 | 352.3 |
| SP | 2011 | 278.4 | 261 |
| SP | 2012 | 69.6 | 284.1 |
| SP | 2013 | 506 | 320.7 |
| SP | 2014 | 515.2 | 333.3 |
| SP | 2015 | 1693.2 | 311.7 |
| SP | 2016 | 457 | 274.2 |
| SP | 2017 | 20.4 | 295.9 |
| SP | 2018 | 42.4 | 305.2 |
| SP | 2019 | 968.2 | 303.6 |
| TO | 2001 | 417.3 | 3548.37 |
| TO | 2002 | 163.9 | 3474.95 |
| TO | 2003 | 153.1 | 3755.25 |
| TO | 2004 | 122.8 | 3769.09 |
| TO | 2005 | 257.1 | 3256.45 |
| TO | 2006 | 446.1 | 3189.43 |
| TO | 2007 | 957 | 2259.77 |
| TO | 2008 | 791 | 1902.78 |
| TO | 2009 | 343.9 | 1877.92 |
| TO | 2010 | 668.8 | 1873.55 |
| TO | 2011 | 855.4 | 1774.83 |
| TO | 2012 | 819.4 | 1788.42 |
| TO | 2013 | 581.5 | 2893.88 |
| TO | 2014 | 244 | 2288.36 |
| TO | 2015 | 527.4 | 3118.68 |
| TO | 2016 | 520.5 | 1640.65 |
| TO | 2017 | 314 | 1721.61 |
| TO | 2018 | 197.8 | 1553.15 |
| TO | 2019 | 936.4 | 1523.75 |

Brazilian states: BA - Bahia, DF – Distrito Federal, GO - Goiás, MA - Maranhão, MG - Minas Gerais, MS - Mato Grosso do Sul, MT - Mato Grosso, PI - Piauí, SP - São Paulo and TO - Tocantins.
