# Supplementary material for: The fewer, the better fare: Can the loss of vegetation in the Cerrado drive the increase in dengue fever cases infection?
Source: PLoS One. 2022 Jan 13;17(1):e0262473. doi: 10.1371/journal.pone.0262473 (PMC8757950; doi:10.1371/journal.pone.0262473)
Supplement: S2 Table — AIC: Akaike information criterion, BIC: Bayesian information criterion, ME: Mean Error, RMSE: Root Mean Square, MAE: Mean Absolute Error, MPE: Mean Percentage Error, MAPE: Mean Absolute Percentage Error, ACF1: first-order partial autocorrelation coefficient. Brazilian states: BA—Bahia, DF–Distrito Federal, GO—Goiás, MA—Maranhão, MG—Minas Gerais, MS—Mato Grosso do Sul, MT—Mato Grosso, PI—Piauí, SP—São Paulo and TO—Tocantins. (DOCX) [file pone.0262473.s002.docx]

**Table S2.** Statistical parameters obtained for the ARIMA model adjusted for dengue fever cases in Brazilian states.

| STATE | ME | RMSE | MAE | MPE | MAPE | ACF1 | AIC | BIC |
| --- | --- | --- | --- | --- | --- | --- | --- | --- |
| BA | 3.5 | 173.1 | 150.3 | -91.3 | 124.5 | 0.0717 | 229.56 | 231.23 |
| DF | 15.0 | 208.6 | 133.0 | -31.3 | 100.2 | -0.4469 | 223.78 | 224.56 |
| GO | -37.3 | 837.4 | 581.9 | -33.2 | 82.0 | -0.3086 | 259.49 | 260.27 |
| MA | 1.0 | 65.8 | 42.9 | -31.3 | 48.8 | -0.1889 | 197.56 | 200.06 |
| MG | 150.2 | 393.8 | 271.7 | 1.5 | 89.8 | -0.2671 | 261.38 | 262.93 |
| MS | -61.1 | 909.0 | 751.0 | -956.4 | 994.6 | 0.0177 | 287.92 | 289.59 |
| MT | 32.7 | 417.8 | 230.1 | -25.3 | 53.3 | -0.0349 | 260.97 | 262.64 |
| PI | 0.4 | 98.3 | 83.1 | -51.7 | 76.2 | 0.0112 | 209.12 | 210.79 |
| SP | 91.1 | 381.6 | 199.9 | -202.9 | 246.9 | -0.1053 | 244.99 | 245.77 |
| TO | 5.0 | 201.1 | 158.9 | -27.4 | 49.4 | 0.1356 | 233.63 | 236.13 |

AIC: Akaike information criterion, BIC: Bayesian information criterion, ME: Mean Error, RMSE: Root Mean Square, MAE: Mean Absolute Error, MPE: Mean Percentage Error, MAPE: Mean Absolute Percentage Error, ACF1: first-order partial autocorrelation coefficient. Brazilian states: BA - Bahia, DF – Distrito Federal, GO - Goiás, MA - Maranhão, MG - Minas Gerais, MS - Mato Grosso do Sul, MT - Mato Grosso, PI - Piauí, SP - São Paulo and TO - Tocantins.
