# Supplementary material for: The fewer, the better fare: Can the loss of vegetation in the Cerrado drive the increase in dengue fever cases infection?
Source: PLoS One. 2022 Jan 13;17(1):e0262473. doi: 10.1371/journal.pone.0262473 (PMC8757950; doi:10.1371/journal.pone.0262473)
Supplement: S3 Table — AIC: Akaike information criterion, BIC: Bayesian information criterion, ME: Mean Error, RMSE: Root Mean Square, MAE: Mean Absolute Error, MPE: Mean Percentage Error, MAPE: Mean Absolute Percentage Error, ACF1: first-order partial autocorrelation coefficient. Brazilian states: BA—Bahia, DF–Distrito Federal, GO—Goiás, MA—Maranhão, MG—Minas Gerais, MS—Mato Grosso do Sul, MT—Mato Grosso, PI—Piauí, SP—São Paulo and TO—Tocantins. (DOCX) [file pone.0262473.s003.docx]

**Table S3.** Statistical parameters obtained for the ARIMA model adjusted for loss of native vegetation in the Cerrado in Brazilian states.

| STATE | ME | RMSE | MAE | MPE | MAPE | ACF1 | AIC | BIC |
| --- | --- | --- | --- | --- | --- | --- | --- | --- |
| BA | -137.3 | 0.03 | 0.02 | -13.3 | 18.1 | -0.17 | 233.4 | 236.5 |
| DF | -0.6 | 15.4 | 8.7 | -19.8 | 69.3 | 0.003 | 144.8 | 146.4 |
| GO | 0.02 | 0.09 | 0.04 | -12.7 | 25.1 | -0.10 | 268.3 | 269.1 |
| MA | -89.0 | 0.05 | 0.04 | -5.3 | 14.4 | -0.15 | 247.2 | 248.0 |
| MG | -118.9 | 583.6 | 398.7 | -12.2 | 39.0 | 0.0008 | 255.3 | 256.9 |
| MS | -40.6 | 0.02 | 0.014 | -7.11 | 26.9 | -0.17 | 222.0 | 223.5 |
| MT | -528.1 | 0.003 | 0.002 | -14.4 | 38.27 | -0.05 | 300.5 | 301.2 |
| PI | 2.15 | 189.0 | 131.4 | -5.45 | 17.2 | -0.01 | 233.3 | 235.8 |
| SP | -1.35 | 52.94 | 34.29 | 4.0 | 155.9 | -0.15 | 167.9 | 168.7 |
| TO | -8.11 | 484.3 | 324.0 | -5.6 | 15.3 | -0.17 | 251.0 | 251.8 |

AIC: Akaike information criterion, BIC: Bayesian information criterion, ME: Mean Error, RMSE: Root Mean Square, MAE: Mean Absolute Error, MPE: Mean Percentage Error, MAPE: Mean Absolute Percentage Error, ACF1: first-order partial autocorrelation coefficient. Brazilian states: BA - Bahia, DF – Distrito Federal, GO - Goiás, MA - Maranhão, MG - Minas Gerais, MS - Mato Grosso do Sul, MT - Mato Grosso, PI - Piauí, SP - São Paulo and TO - Tocantins.
